# Promising biocontrol effects of a native hemiparasitic plant against a non-native C4 grass

**DOI:** 10.1038/s41598-026-44801-0

**Published:** 2026-03-20

**Authors:** Csaba Tölgyesi, Alida Anna Hábenczyus, Fanni Molnár, Kata Anna Bán, Ádám Lőrincz, Kata Frei, Zoltán Bátori, László Erdős, Zalán Czékus, Attila Ördög, Klára Terézia Kovács, Péter Török, Péter Poór

**Affiliations:** 1 MTA-SZTE ‘Momentum’ Applied Ecology Research Group, University of Szeged, Szeged, Hungary; 2 Department of Ecology, University of Szeged, Szeged, Hungary; 3 HUN-REN Centre for Ecological Research, Institute of Ecology and Botany, Lendület Seed Ecology Research Group, Vácrátót, Hungary; 4 Department of Plant Biology, University of Szeged, Szeged, Hungary; 5 HUN-REN-UD Functional and Restoration Ecology Research Group, University of Debrecen, Debrecen, Hungary; 6 Department of Ecology, University of Debrecen, Debrecen, Hungary; 7 Botanical Garden-Center for Biological Diversity Conservation in Powsin, Polish Academy of Sciences, Warszawa, Poland

**Keywords:** Biocontrol, *Festuca vaginata*, Invasive species, Non-native host, *Odontites luteus*, Plant-plant interaction, *Sporobolus cryptandrus*, Ecology, Ecology, Plant sciences

## Abstract

Hemiparasitic plants are known to be able to diversify degraded grasslands by lowering the competitive power of dominant grasses. Recent research indicates that hemiparasites may also be used against invasive alien plants. Here, we tested the effects of *Odontites luteus*, a native European hemiparasite, on *Sporobolus cryptandrus*, a recently established and rapidly spreading C4 grass of North-American origin. We found that *Odontites* considers *Sporobolus* a suitable host and reduces its biomass production (and potentially its competitive ability) by approx. 50%, equaling the effect on its major native host, *Festuca vaginata.* However, *Festuca* showed severe metabolic impairment (reduced photosynthetic capacity and increased physiological stress) under hemiparasite pressure. So, the application of hemiparasites is a promising biocontrol tool against *Sporobolus* (and potentially other invasive C4 grasses), but it is not a silver bullet. We cannot expect a full eradication of the invasive species and a recovery of the native community but thinning monodominant *Sporobolus* stands to allow certain populations of native species, particularly those resistant to *Odontites*, to come back is a more realistic goal. This could lead to a partial recovery of the former species composition and an improvement of ecosystem functions, such as providing food for pollinators.

**Supplementary Information:**

The online version contains supplementary material available at 10.1038/s41598-026-44801-0.

## Introduction

Biological invasion is one of the main threats to global biodiversity and the provisioning of ecosystem services to humans^1–4^, and regarding species endangerment, it is second in importance only to direct habitat destruction^5^. Among invasive plants, the two major groups that pose global challenges for nature conservation and overall human livelihood are grasses and woody species^6^. Their rapid spread can be a consequence of various circumstances, such as functional incompleteness in the host community or superior trait combinations of the species, including effective reproduction and dispersal strategies, high competitive ability and few enemies^7,8^. *Sporobolus cryptandrus* (hereafter referred to as *Sporobolus*), a North-American perennial bunchgrass is one such species. It was first recorded in Hungary, Central Europe, in 2016^9^ and is now spreading rapidly in several hundred individual locations of dry sand steppe habitats^10^. It has tough leaves, which are generally avoided by grazers in the non-native range, has an extensive root system lending efficient resource acquisition capacity, and its seeds can become dominant in the soil seed bank within a few years after colonization^11^. On top of this, it has C4 photosynthetic metabolism, which, under ongoing climate change, featuring increasingly dry and warm summers in the region^12^, provides great advantage in the host plant community, dominated by C3 species. In line with these, Hábenczyus et al.^13^ showed that *Sporobolus* is a transformer species, having stronger suppressive effects on the host vegetation in the non-native Hungarian range than in its native North American range.

Controlling *Sporobolus* in Hungary is a top issue in nature conservation but, at the same time, is a great challenge, as conventional methods are unlikely to be efficient. Chemical control is detrimental for native species too, while we can expect quick secondary invasion from the soil seed bank after the eradication from the above-ground vegetation, as observed in the case of other invasive herbaceous species with good dispersal strategies^14,15^. Mechanical management, such as mowing, is also challenging due to the erosion-prone structure of the dry sandy substrate, favoured by the species^16,17^. Another, so far unstudied approach is the use of biocontrol agents.

The idea behind the classical biological control is the introduction of a natural parasite, predator or pathogen of the invasive species in order to suppress its non-native population, as the enemy release hypothesis^18^ suggests. However, these secondary introductions potentially give rise to a new invasion^19^. The biotic resistance hypothesis^20^ states, that native members of the recipient community are also capable of restraining non-native stands of species. Applying species native to the invaded area as biocontrol agents excludes the chance of a new invasion, furthermore, it is a method proved to be effective against expansive native species^21^. Parasitic and hemiparasitic plants attach to the vascular system of the host plant, thus achieving direct nutrient extraction, which results in a direct reduction in the host’s biomass, and also can have a negative effect on photosynthesis-related metabolism^22^. Lower photosynthetic activity decreases the host’s growing rate, which ultimately leads to a biomass deficit^23^. It has been shown that native hemi-parasitic species can efficiently suppress over-abundant native grasses. For instance, sowing *Rhinanthus alectorolophus* in degraded habitats dominated by *Calamagrostis epigejos* is an emerging method to restore former, species-rich grasslands^24,25^.

The most abundant and widespread hemi-parasitic plant of dry sandy steppes where *Sporobolus* appeared as an invasive alien species, is *Odontites luteus* (hereafter *Odontites*), an annual forb of the Orobanchaceae family. Its native hosts include perennial C3 grasses; it germinates late in the growing season and blooms mainly in August and September^26^, so its phenology largely overlaps with that of *Sporobolus*^27^. However, resistance among plants to hemi-parasitic species does occur^28^, and the magnitude of the effects of hemiparasites even on preferred hosts can vary greatly^29^.

Therefore, in this study, we aimed to quantify the effect of *Odontites* on *Sporobolus* in a controlled mesocosm experiment as a first step to assess its applicability in the wild. Specifically, we measured above-ground biomass production and various physiological parameters indicative of photosynthetic activity (chlorophyll and RUBISCO content) and stress level (anthocyanin, electrolyte leakage, and lipid-peroxidation) in *Sporobolus* with and without *Odontites*, and compared these to the effects of *Odontites* on a common native host, *Festuca vaginata* (hereafter *Festuca*). Furthermore, we also evaluated the competitive effects between the two grasses by growing them together, with or without *Odontites*.

## Results

### Biomass

**Fig. 1:**
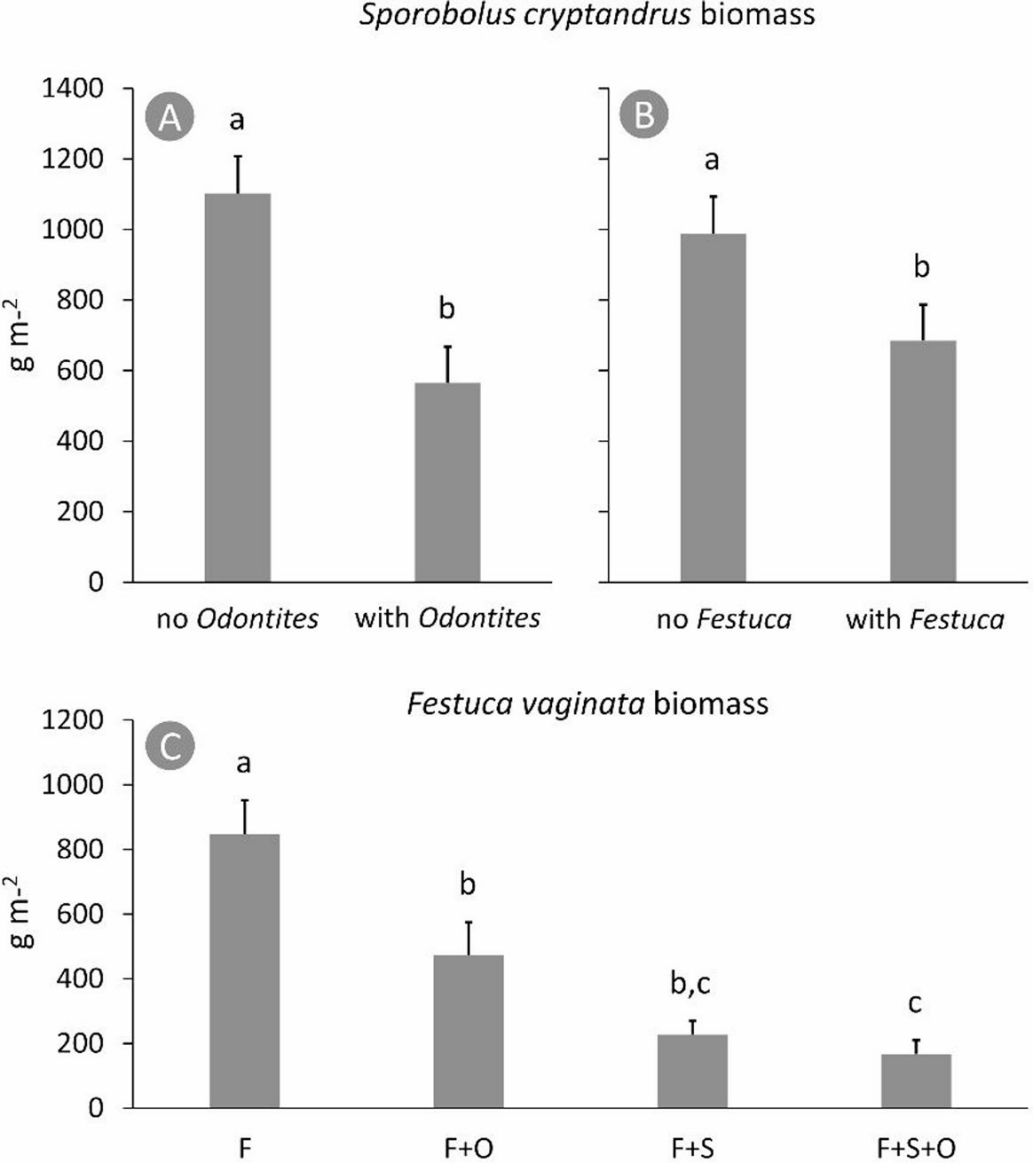
Effects of *Odontites luteus* and *Festuca vaginata* on the dry biomass production of *Sporobolus cryptandrus* (**A** and **B**, respectively), and the effect of *Odontites luteus* and *Sporobolus cryptandrus* on the biomass production of *Festuca vaginata* (**C**). Pairwise comparisons of all category combinations are shown in subplot (**C**) because of a significant interaction between the effects of the parasite and the other grass. Different lower case letters indicate significant differences (*p* < 0.05); whiskers represent standard error of the mean. F: *Festuca vaginata*, O: *Odontites luteus*, S: *Sporobolus cryptandrus.*

*Odontites* considered both *Sporobolus* and *Festuca* as suitable hosts and developed vital stands in all flowerbeds, with many individuals reaching the generative stage by the end of the growing season. Its biomass was not affected by host species identity (F = 0.30, *p* = 0.746) and reached an average value of 311.8 ± 39.0 g m^− 2^ (mean ± standard deviation). *Odontites* reduced the biomass of *Sporobolus* by 48.6% (F = 27.85, *p* < 0.001), while *Festuca* caused a 29.4% reduction in it (F = 8.16, *p* = 0.009) (Fig. 1A-B), and there was no interaction between their effects (F = 0.18, *p* = 0.675).

The biomass of *Festuca* was significantly reduced by both *Odontites* (F = 8.15, *p* = 0.010) and *Sporobolus* (F = 35.12, *p* < 0.001) by 42.3% and 72.0%, respectively. The interaction was marginally significant among the two effects (F = 3.36, *p* = 0.082), so we decided to perform all pairwise comparisons of category combinations, and found that *Odontites* had no further significant negative effect on *Festuca* in flowerbeds where *Sporobolus* was also present (Fig. 1C; Table 1).

**Table 1:** Results of pairwise comparisons of *Festuca vaginata* biomass among all treatment combinations, calculated with Tukey’s HSD test. Significant differences (*p* < 0.05) are marked with boldface.

Category combination	*p*-value
F vs. F + O	**0.017**
F vs. F + S	**< 0.001**
F vs. F + S+O	**< 0.001**
F + O vs. F + S	0.165
F + O vs. F + S+O	**0.041**
F + S vs. F + S+O	0.887

### Photosynthetic activity

**Fig. 2:**
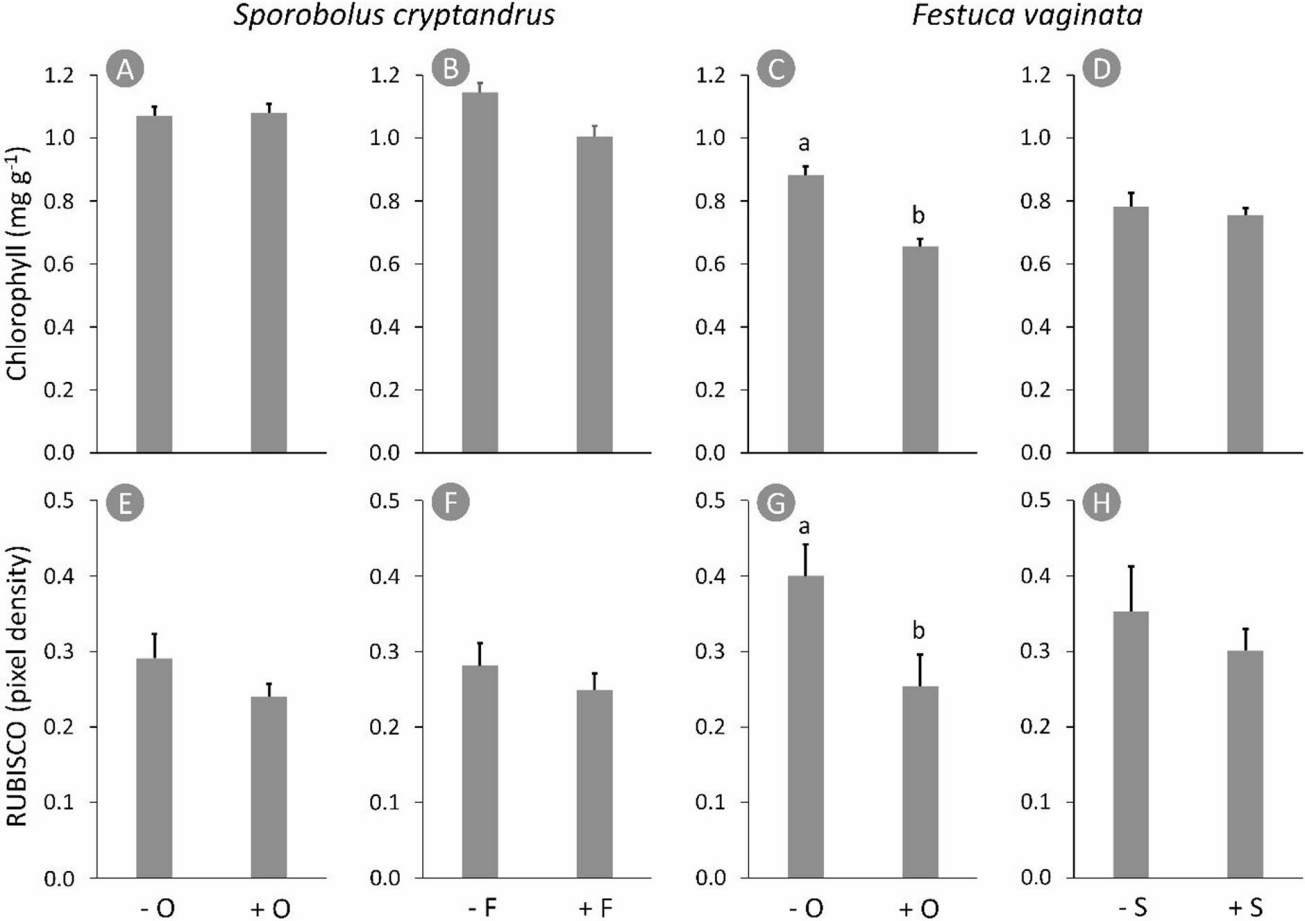
Changes in the photosynthetic capacity of *Sporobolus cryptandrus* and *Festuca vaginata* in the presence of *Odontites luteus* or the other grass. Quantification is based on six individual gels with no lanes rearranged or spliced. Original full-length uncropped blots are provided in Supplementary Figs. 1–6. Different lower case letters indicate significant differences (*p* < 0.05); the other pairs do not differ significantly. Whiskers represent standard error of the mean. − O: without *Odontites luteus*, + O: with *Odontites luteus*, − F: without *Festuca vaginata*, + F: with *Festuca vaginata*, − S: without *Sporobolus cryptandrus*, + S: with *Sporobolus cryptandrus*.

Regarding chlorophyll content in the leaves of *Sporobolus*, neither *Festuca*, nor *Odontites* had any detectable effect and the interaction was also non-significant (F < 3.02, *p* > 0.120) (Fig. 2A-B). In contrast, *Odontites* significantly reduced the chlorophyll content in the *Festuca* leaves, by 25.8% (F = 15.70, *p* = 0.004), but *Sporobolus* had no effect (F = 0.16, *p* = 0.699), and we found no interaction either (F = 3.47, *p* = 0.088) (Fig. 2C-D). RUBISCO content in the *Sporobolus* leaves was not affected by the other species (F < 2.36, *p* > 0.143) (Fig. 2E-F, Fig. S1-S3). RUBISCO in *Festuca* leaves decreased in the presence of *Odontites* by 36.5% (F = 5.96, *p* = 0.026), although *Sporobolus* had no detectable effect (F = 0.76, *p* = 0.397) and there was no detectable interaction (F = 0.83, *p* = 0.406) (Fig. 2G-H, Fig. S4-S6).

### Physiological stress

**Fig. 3:**
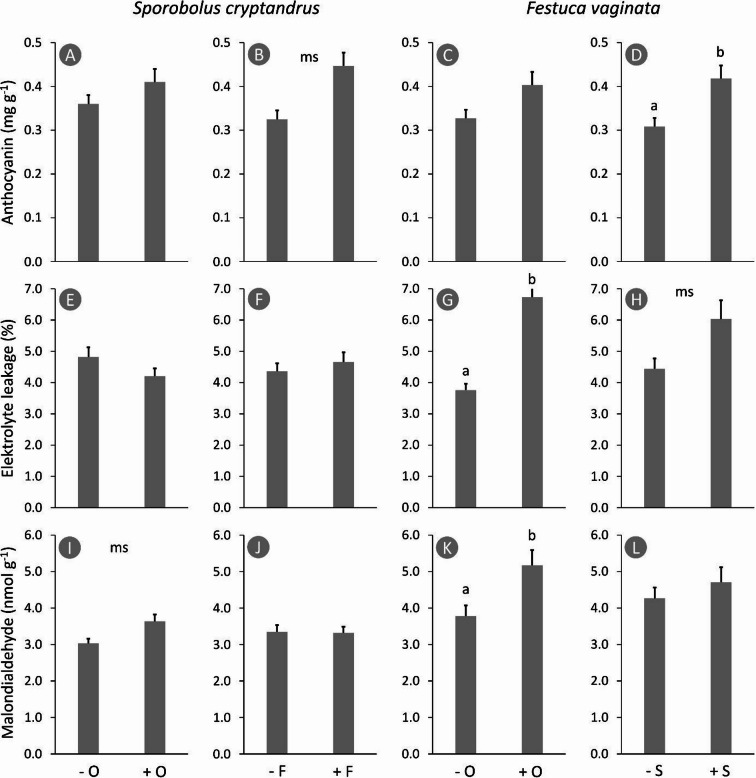
Changes in the physiological stress level in *Sporobolus cryptandrus* and *Festuca vaginata* leaves in the presence of *Odontites luteus* or the other grass. Different lower case letters indicate significant differences (*p* < 0.05); the other pairs do not differ significantly. Whiskers represent standard error of the mean. − O: without *Odontites luteus*, + O: with *Odontites luteus*, − F: without *Festuca vaginata*, + F: with *Festuca vaginata*, − S: without *Sporobolus cryptandrus*, + S: with *Sporobolus cryptandrus*, ms: marginally significant.

The accumulation of anthocyanin is considered an adaptive response of plants to various biotic and abiotic stress conditions. As potent antioxidants, anthocyanins contribute to the scavenging of reactive oxygen species (ROS), thereby reducing oxidative damage and enhancing stress tolerance. Anthocyanin concentrations in the *Sporobolus* leaves were not affected by *Odontites* (F = 0.67, *p* = 0.437) but we detected a marginally significant increase of 37.6% when grown together with *Festuca* (F = 3.82, *p* = 0.087); we found no significant interaction in the model (F = 0.31, *p* = 0.595) (Fig. 3A-B). *Odontites* did not affect anthocyanin content in *Festuca* leaves (F = 2.61, *p* = 0.144), but *Sporobolus* increased it by 35.7% (F = 5.85, *p* = 0.037), and there was no interaction (F = 2.33, *p* = 0.158) (Fig. 3C-D).

*Sporobolus* showed no increase in electrolyte leakage in the presence of its competitor and/or parasite (F < 0.65, *p* > 0.445) (Fig. 3E-F). In contrast, *Odontites* triggered an increase of 78.9% in electrolyte leakage in *Festuca* leaves (F = 15.49, *p* = 0.004), and even *Sporobolus* caused a marginally significant increase of 35.7% (F = 3.95, *p* = 0.082); interaction could not be detected between the two predictors (F = 0.16, *p* = 0.698) (Fig. 3G-H).

MDA content, which is an indicator of lipid peroxidation and membrane damage caused by oxidative stress, showed a marginally significant increase of 20.1% in *Sporobolus* leaves when infected by *Odontites* (F = 5.07, *p* = 0.054), but *Festuca* had no effect (F = 0.01, *p* = 0.922), and no interaction was detected between the predictors (F = 0.67, *p* = 0.436) (Fig. 3I-J). *Odontites* increased MDA in *Festuca* leaves by 25.3% (F = 6.22, *p* = 0.047), but *Sporobolus* had no effect (F = 1.61, *p* = 0.252), and no interaction was detected between the predictors (F = 0.14, *p* = 0.719) (Fig. 3K-L).

## Discussion

In this study we aimed to assess the potential of a hemiparasitic plant, *Odontites luteus*, to suppress *Sporobolus cryptandrus*, an invasive alien grass spreading in dry sandy grasslands of Central Europe. In a mesocosm design we found that *Odontites* recognizes *Sporobolus* as an equally good host as one of its main native hosts, *Festuca vaginata*. Resource removal by the parasite nearly halved the biomass production of *Sporobolus*, which is in line with many studies showing that the parasite-host interaction can greatly reduce growth rates^30,31^. The loss of resources in the host can cause reduced photosynthetic capacity and other metabolic impairment^31,32^, but here we found little physiological strain in *Sporobolus*. The indicators of photosynthetic capacity remained unaffected and among the stress indicators we found only a marginally significant increase in MDA, a marker of lipid peroxidation, indicating mild, manageable oxidative stress in the host plant, while the other indicators, e.g. the level of anthocyanin, an important antioxidant, did not change under parasite pressure.

The effect of *Odontites* on the biomass production of *Festuca* was similar in magnitude to that on *Sporobolus* (42.3% and 48.6%, respectively), but this was also accompanied by a profound metabolic response, including impaired photosynthetic capacity and increased physiological stress. This is indicated by significantly higher electrolyte leakage and MDA content, which correlates with enhanced oxidative stress and disruption of membrane integrity likely due to ROS accumulation and localized tissue damage. The reason why a similar decrease of biomass in the hosting grasses came with a higher cost in *Festuca* may lie in the higher resource use efficiency of *Sporobolus* due to its C4 metabolism^9^. In other words, our findings suggest that producing the same amount of biomass by the studied C3 grass under parasite pressure strains its metabolism more than that of the C4 invader, although other traits of the species may also have a role.

The high resource use efficiency of *Sporobolus* may also be a major factor in its invasive ability in the host plant communities^33^, where it can outcompete native species^13^. Likewise, our findings also indicated that *Sporobolus* is competitively superior to *Festuca*, because, when grown together (without a parasite to complicate the interaction), the biomass of *Festuca* dropped by about three quarters, while that of *Sporobolus* just slightly decreased. The negative effect of *Sporobolus* on *Festuca* was also indicated by increased stress levels, e.g. by elevated anthocyanin content, which suggests either interspecific competition or subtle stress signals triggered by the presence of the neighbouring plant. However, when the parasite was also added (i.e. the *Festuca*+*Sporobolus*+*Odontites* category), it did not decrease the biomass of *Festuca* any further than what we detected in the *Festuca*+*Sporobolus* treatment category (see Fig. 1C). The explanation may lie in that hemiparasites prefer tapping on dominant species, which are abundant in resources^10,34^. In a setting where *Sporobolus* and *Festuca* coexist, and more resources are allocated to *Sporobolus*, while *Festuca* is resource-deprived, *Odontites* may prefer *Sporobolus* to *Festuca*. So, the balanced effect that we detected in the parasite–single host categories got biased for *Sporobolus*, and *Festuca* could at least partially be released from the parasite pressure. As the detailed process of this mechanism still leaves questions, it may be tested in the future by evaluating host–parasite connections (e.g. haustorium densities;^35^ in the different interaction categories. Alternatively, *Odontites* could reduce the competitive power of *Sporobolus* so much that it offset the effect of *Odontites* on *Festuca*. This mechanism does not necessarily require differential host preference, however, also needs to be unravelled.

Whichever explanation has the higher importance; it is clear that *Odontites* is a promising tool to tackle *Sporobolus* invasion but is not a silver bullet. Těšitel et al.^10^ claimed that parasites should have stronger effects on invasive alien species than on native species to be considered effective biocontrol agents. For instance, the Australian *Cassytha pubescens* has stronger effects on the invasive *Ulex europaeus* than on the native *Leptospermum myrsinoides*^36^, and the Chinese *Cuscuta chinensis* also suppresses the invasive *Bidens pilosa* more than the congeneric native *B. bipinnata*^37^. However, in our case, the native species is more vulnerable to the effects of the parasite than the invasive species, so the fact that *Sporobolus* is naïve to *Odontites* does not give enough leverage for a stronger effect. But what can be achieved then in the wild with the application of *Odontites*?

Based on the present findings we can reasonably expect that the application of *Odontites* does not act synergistically to that of *Sporobolus* on native grasses, allowing them to survive in small populations, such as in the *Sporobolus*+*Festuca*+*Odontites* treatment category. Furthermore, other native species, which are competitively excluded by *Sporobolus* (see^13^, but are not preferred by *Odontites* can have good chances to come back. Although *Odontites* species have broad host species spectra, non-leguminous forbs are generally less preferred than grasses^35,38^. For example, some Asteraceae species are capable of encapsulating hemiparasite haustoria before they could become functional, and Plantaginaceae species respond to approaching hemiparasite roots by excessive cell death, precluding the formation of the parasite-host interface necessary for resource transfer^28^. The invaded dry sandy grasslands in Hungary are rich in both Asteraceae (e.g. *Artemisia campestris*, *Solidago virgaurea*, and *Tragopogon floccosus*) and Plantaginaceae (e.g. *Plantago indica* and *P. lanceolata*) species, so they could possibly take advantage of using *Odontites* as a biocontrol agent. However, these assumptions need to be tested with field experiments before introducing *Odontites* as a practical tool. This is also needed because there is evidence that some hemiparasitic species can facilitate invasion by creating gaps in the vegetation, which can be filled by colonizing invasive species^39^. In large, homogeneous stands of *Sporobolus* in Hungary, the creation of gaps of any kind, even if partially recolonized by the same invasive species later on, is a desirable outcome. Even if no native species return, *Odontites*, being an insect-pollinated plant blooming in late summer, restores an important ecosystem function, which is absent in monodominant stands of *Sporobolus*, pollinated by wind^13^. In sparsely invaded patches though, *Odontites* may have more nuanced effects, and its application as prevention in areas at risk of future invasion is particularly questionable and needs careful testing. So, to sum up, *Odontites luteus* has the potential to be used as a biocontrol agent against *Sporobolus cryptandrus*, although not to eradicate it but to thin its populations to allow for a partial recovery of the native species composition and ecosystem functions.

## Materials and methods

### Study species

*Sporobolus cryptandrus* (Torr.) A. Gray (Poaceae) is a long-lived perennial bunchgrass, native throughout North America, including Southern Canada, the USA and the Northern part of Mexico^40–42^. Due to its efficient water uptake and C4 photosynthetic pathway, *Sporobolus* is considered to be one of the most drought resistant species in American short-grass prairie^43^. In its native range, *Sporobolus* stems are able to withstand heavy disturbance due to their protected root crown, late maturity and because they are less preferred by grazers than other species^41^. However, it can be killed by overgrazing as a result of continued close cropping^44^. *Sporobolus* has been reported as non-native from Australia, Japan, New Zealand, and Argentina^46–48^, and has appeared in isolated locations throughout Eurasia^48–54^. *Sporobolus* established permanent stands mostly in the European Mediterranean and in the semi-arid regions of Eastern Central and Eastern Europe^9^.

*Festuca vaginata* Waldst. & Kit. ex Willd. (Poaceae) is a native perennial C3 bunch grass species, widespread and abundant in open calcareous sandy grasslands in Hungary. It tolerates nutrient-poor and dry conditions, as well as moderate grazing^55^.

*Odontites luteus* (L.) Clairv. (Orobanchaceae) is a native and common annual forb of Hungarian dry sandy steppes. Being a hemiparasite, *Odontites* is able to perform functional photosynthesis, while obtaining mineral nutrients and water from its host via haustorial root connections attached to the host’s xylem^56^. *Odontites* requires periods of low temperature to initiate germination, which ensures that most plant species are dormant at the time, thus the hemiparasite’s seedlings avoid above-ground competitive pressure from the surrounding vegetation^57^. *Odontites* has a wide range of potential host species, and as such it does not need specific chemical inductions of the host to stimulate its germination^58^. Although it may reach high densities locally, *Odontites* has not been reported to cause harmful effects at the community level regarding species diversity.

Voucher specimens of each studied species were deposited in the public herbarium of the Department of Ecology, University of Szeged. The specimens were identified by Csaba Tölgyesi.

### Mesocosm design

**Fig. 4:**
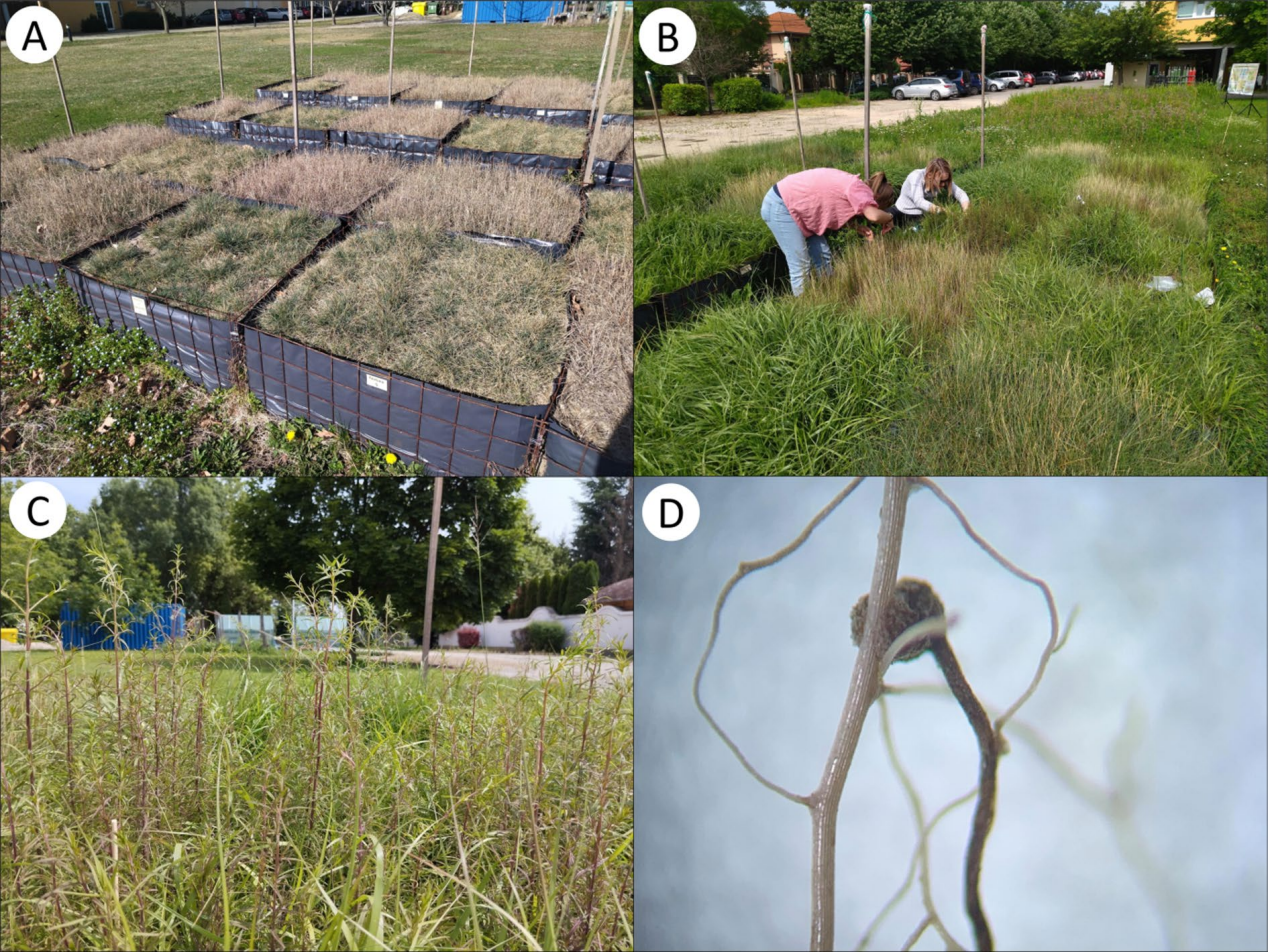
Flowerbeds with *Sporobolus cryptandrus*, *Festuca vaginata* and their combination in spring 2023, before the germination of *Odontites luteus* (**A**); maintenance work of the fully grown vegetation in the flowerbeds in summer 2023 (**B**); vital stems of *Odontites luteus* parasitizing *Sporobolus cryptandrus* (**C**); and a haustorium of *Odontites luteus* (dark nodule) on *Sporobolus cryptandrus* (light brown root) (**D**).

We prepared a total of 36 raised flowerbeds with dimensions of 1 m × 1 m × 0.5 m (width, length, and depth). We filled them with commercial sand mined in central Hungary, so it is the same coarse-grained calcareous substrate on which the wild populations of the studied plant species grow, although it was poorer in nutrients as it was excavated from deep layers^59^. Therefore, we used nitrogen fertilizer in commercially available granules (6.0 g m^–2^) once after sowing *Festuca* and *Sporobolus*. We randomly allocated one of the following six categories to the flowerbeds, leading to six repetitions for each of them: (1) *Sporobolus* only, (2) *Sporobolus* + *Odontites*, (3) *Festuca* only, (4) *Festuca* + *Odontites*, (5) *Sporobolus* + *Festuca*, and (6) *Sporobolus* + *Festuca* + *Odontites*. In spring 2022, we sowed *Sporobolus* and *Festuca* with a total seed density of 2000 seeds m^− 2^ in their respective seedbeds. In summer 2022, we thinned the populations by placing a grid with a mesh size of 0.1 m × 0.1 m over the seedbeds and leaving only two grass stems in every grid cell (two *Sporobolus*, two *Festuca*, or one *Sporobolus* and one *Festuca*). Thus, we got largely homogeneous vegetation cover in every flowerbed (Fig. 4A-B). In October, 2022 we sowed *Odontites* in the respective flowerbeds with a density of 1000 seeds m^− 2^. *Odontites* successfully germinated and developed vital, flowering stands by the end of the summer of 2023 (Fig. 4C-D). All sown seeds were hand-collected in 2022, near the town of Kiskunhalas, Hungary (N46.392, E19.552). Flowerbeds were regularly weeded and regularly watered (once a week in rain-free periods) during the growing seasons of 2022 and 2023.

### Data collection

We measured biomass production of the studied species by clipping all aboveground plant material in 0.5 m × 0.5 m quadrats in every seedbed in August 2023. We then dried the samples at 40 °C in a drying chamber, sorted according to species, and measured them gravimetrically.

We assessed the vitality of the two host grasses (*Sporobolus* and *Festuca*) by measuring physiological parameters indicative of photosynthetic activity (chlorophyll *a* and *b* content) and stress level^60^. Anthocyanins are flavonoid-derived pigments that can be found in flowers, leaves, and fruits, where they contribute to coloration and play important protective roles. Anthocyanins are widely-recognized sensitive markers of stress as their accumulation is generally induced by adverse abiotic (e.g. high light, drought, cold) and biotic stress (e.g. fungal infection, herbivory) conditions, reflecting stress-related physiological changes in plant tissues^61–63^. Beyond serving as indicators, anthocyanins function as potent antioxidants that scavenge ROS, whose overaccumulation under stress can inhibit growth and trigger programmed cell death^64^. These functions place anthocyanins in close association with other physiological stress markers such as malondialdehyde (MDA) levels as well as electrolyte leakage^64^. For determining pigment contents, we collected five undamaged leaves from five grass stems of each species from three randomly chosen flowerbeds of each category before biomass clipping. Leaves from the same stem were pooled, minced, and 25 mg of them was homogenized in 1 mL of 100% (v/v) ice-cold acetone. Pigment extraction was performed at 4 °C for 24 h in the dark. Next day, the samples were centrifuged (12,000 × *g*, 15 min, 4 °C), then the pellet was re-extracted with 1 mL of ice-cold acetone/Tris buffer solution (80:20 v/v; pH: 7.8) at 4 °C for 24 h in the dark. Next day, samples were centrifuged again (12,000 × *g*, 15 min, 4 °C), then merged supernatants were used for spectrophotometric anthocyanin and chlorophyll *a* and *b* content determination at 537, 647, and 663 nm (KONTRON, Milano, Italy). We used the following equations for calculating the pigment contents^65^.

$$\begin{gathered} {\mathrm{Anthocyanin}}=0.0{\mathrm{8173}}*{{\mathrm{A}}_{{\mathrm{537}}}} - 0.00{\mathrm{697}}*{{\mathrm{A}}_{{\mathrm{647}}}} - 0.00{\mathrm{2228}}*{{\mathrm{A}}_{{\mathrm{663}}}} \hfill \\ {\mathrm{Chlorophyll}}a=0.0{\mathrm{1373}}*{{\mathrm{A}}_{{\mathrm{663}}}} - 0.000{\mathrm{897}}*{{\mathrm{A}}_{{\mathrm{537}}}} - 0.00{\mathrm{3}}0{\mathrm{46}}*{{\mathrm{A}}_{{\mathrm{647}}}} \hfill \\ {\mathrm{Chlorophyll}}b=0.0{\mathrm{24}}0{\mathrm{5}}*{{\mathrm{A}}_{{\mathrm{647}}}} - 0.00{\mathrm{43}}0{\mathrm{5}}*{{\mathrm{A}}_{{\mathrm{537}}}} - 0.00{\mathrm{55}}0{\mathrm{7}}*{{\mathrm{A}}_{{\mathrm{663}}}} \hfill \\ \end{gathered}$$

We determined the quantity of the large (catalytic) subunit of RUBISCO (RbcL), a major component of carbon fixation, with Western blot analysis following Czékus et al.^65^. The quantity of RbcL correlates with the amount of total RUBISCO protein; therefore, it is suitable for determining the amount of the total protein. For this analysis, we collected five leaves from one grass stem from each species from every flower bed before biomass clipping. We homogenized samples from each stem in liquid nitrogen to get a fine powder, then proteins were extracted using Lacus buffer with some modifications [25 mM Tris–HCl, pH: 7.8, 75 mM NaCl, 15 mM EGTA, 10 mM MgCl_2_, 1 mM dithiothreitol (DTT), 0.5 mM phenylmethylsulfonyl fluoride (PMSF), 0.05% Triton X-100]^66^. Denaturated protein extract (5 µL) was separated on 12% SDS-PAGE, then transferred onto PVDF membrane at 200 mA for 70 min (Immobilon-P, Millipore, USA). After blocking the membrane for 1 h at room temperature with TBS-T buffer [50 mM Tris-HCl, pH: 8.0, 150 mM NaCl, 0.05% Tween 20, 24 mg mL^− 1^ bovine serum albumin (BSA)], the blot was incubated with anti-RbcL (AS03 037, 1:10000) primary (rabbit) antibody (dissolved in TBS-T buffer) at 4 °C overnight. We then washed the membrane three times and incubated it in HRP-conjugated secondary (goat-anti-rabbit) IgG antibody solution (AS09 602, 1:12000) for 1 h at room temperature. After washing four times, RbcL proteins were detected on the membrane using Western Chemiluminescent HRP Substrate (Immobilon, Millipore, USA). We used an A C-DiGit Western blot scanner system (LI-COR Biotechnology, Lincoln, NE, USA) to detect the chemiluminescent signal. All antibodies were purchased from Agrisera (Vännäs, Sweden).

Lipid peroxidation of cell membranes as a consequence of membrane damage (another measure of plant stress) was estimated by determining malondialdehyde (MDA) content, following Ederli et al.^67^. Again, we collected undamaged leaves from five grass stems of each species from the three most representative beds of each category before biomass clipping. Leaves from the same stem were pooled, homogenized with liquid nitrogen, and 100-mg amounts were further ground in 1 mL of 0.1% TCA and 0.1 mL of 4% butylated hydroxytoluene (BHT), then centrifuged at 12,000 × *g* at 4 °C for 20 min. After this, 0.25 mL of the supernatant was added to 1 mL of 20% TCA containing 0.5% thiobarbituric acid (TBA) and incubated at 98 °C for 30 min. We measured MDA content using spectrophotometry at 532 nm, which was corrected with the non-specific absorbance measured at 600 nm (KONTRON, Milano, Italy). MDA content was calculated using an extinction coefficient of 155 mM^− 1^ cm^− 1^. All chemicals were purchased from Sigma-Aldrich (St. Louis, MO, USA).

We determined electrolyte leakage (a measure of cell viability and stress level) according to Sun et al.^68^. We collected a 50-mg undamaged leaf from five grass stems of each species from the three most representative beds of each category before biomass clipping. We incubated the samples in ultrapure distilled water (20 mL) immediately after sampling for 2 h at 24 °C in the dark. We first measured water conductivity after the 2-h incubation (C1) with a conductivity meter (HANNA Instruments, Woonsocket, RI, USA), then total conductivity (C2) after boiling the samples for 40 min. EL was expressed in percentages using the following formula: EL = C1 / C2 × 100.

### Statistical analysis

We first compared the biomass of *Odontites* among the categories it occurred in using one-way analysis of variance, with three levels (*Odontites* + *Sporobolus*, *Odontites* + *Festuca*, and *Odontites* + *Sporobolus* + *Festuca*). Since the grasses (*Sporobolus* and *Festuca*) were sown on their own, too, we applied two-way ANOVAs for each of them, using the presence of the other grass and *Odontites* as explanatory variables (‘present’ or ’absent’) with interaction. If we found significant interaction, we proceeded with comparing all category combinations using Tukey’s HSD test.

For the physiological parameters of *Sporobolus* and *Festuca*, we used linear mixed-effects models. The fixed factors were the presence of the other grass and *Odontites* with interaction, while the random factor was either flowerbed identity (for chlorophyll, anthocyanin, lipid peroxidation, and electrolyte leakage) or Western blot gel (for RUBISCO).

All statistical tests were undertaken in R version 4.2.2^69^. ANOVA was prepared using the *aov* function, and the significance of the explanatory variables was tested with the *anova* function. Tukey’s HSD tests were calculated with the *TukeyHSD* function. Linear mixed-effects models were prepared using the *lmer* function of the ‘lme4’ package^70^. We tested the fixed factors for significance with the *anova* function, supported by the ‘lmerTest’ package^71^.

## Supplementary Information

Below is the link to the electronic supplementary material.

Supplementary Material 1

## Data Availability

All data used in the study will be made available in Dryad Data Repository upon acceptance of the paper. The datasets generated during and/or analysed during the current study are also available from the first author (tolgyesi.csaba@bio.u-szeged.hu) on reasonable request.
