# Supplementary material for: Promising biocontrol effects of a native hemiparasitic plant against a non-native C4 grass
Source: Sci Rep. 2026 Mar 20;16:14341. doi: 10.1038/s41598-026-44801-0 (PMC13144604; doi:10.1038/s41598-026-44801-0)
Supplement: Supplementary file 1 — Supplementary Material 1 [file 41598_2026_44801_MOESM1_ESM.docx]

Supplementary material to the paper

**Promising biocontrol effects of a native hemiparasitic plant against a non-native C4 grass**

*Scientific Reports*


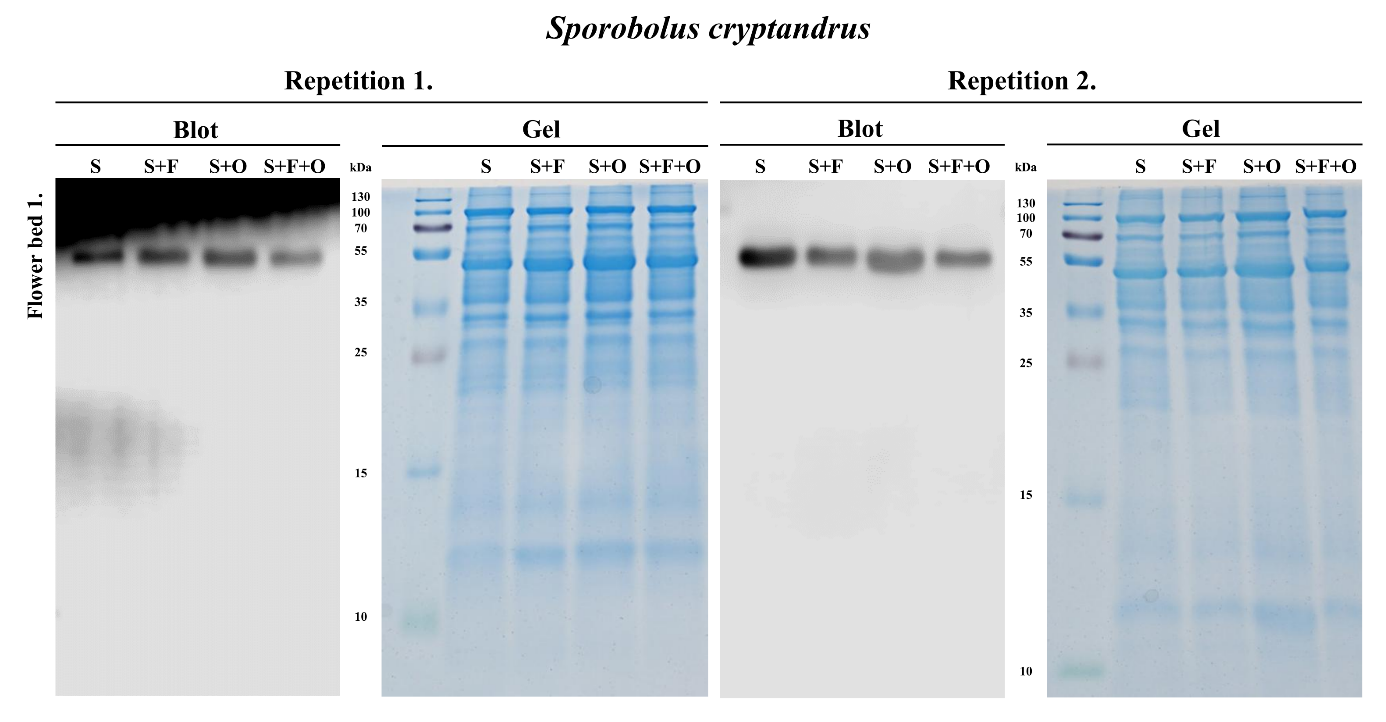


**Figure S1** Western blot gels of RUBISCO from *Sporobolus cryptandrus* leaves with and without the hemiparasite and its native competitor. F: *Festuca vaginata*, O: *Odontites luteus*, S: *Sporobolus cryptandrus*. Neither groups differed significantly from each other (see statistics in the main text).


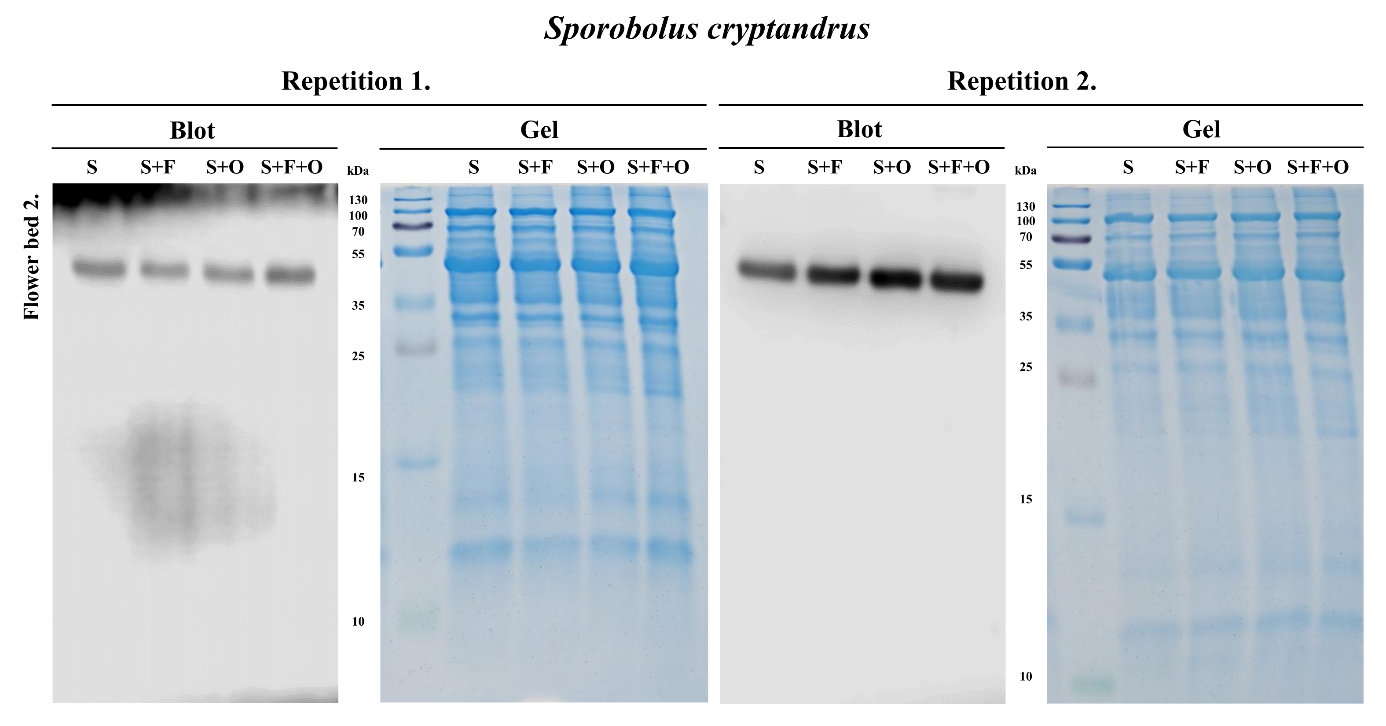


**Figure S2** Western blot gels of RUBISCO from *Sporobolus cryptandrus* leaves with and without the hemiparasite and its native competitor. F: *Festuca vaginata*, O: *Odontites luteus*, S: *Sporobolus cryptandrus*. Neither groups differed significantly from each other (see statistics in the main text).


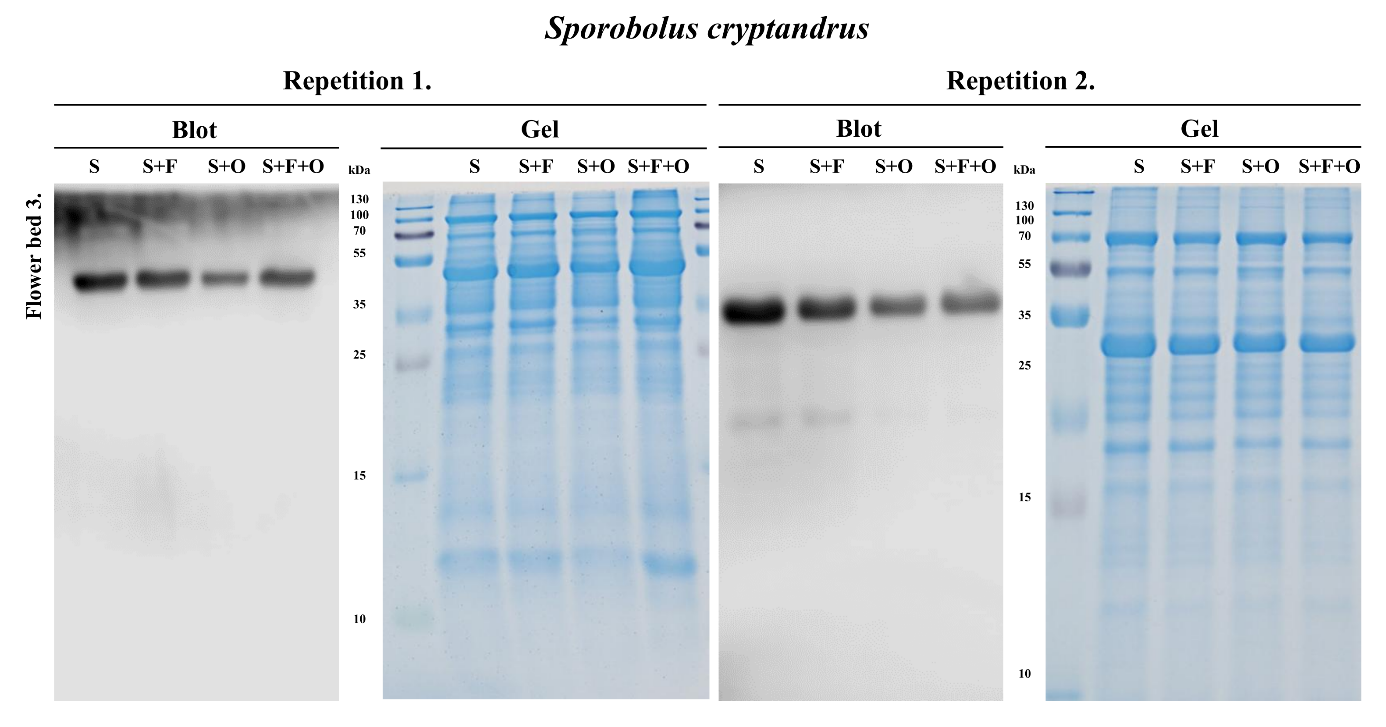


**Figure S3** Western blot gels of RUBISCO from *Sporobolus cryptandrus* leaves with and without the hemiparasite and its native competitor. F: *Festuca vaginata*, O: *Odontites luteus*, S: *Sporobolus cryptandrus*. Neither groups differed significantly from each other (see statistics in the main text).


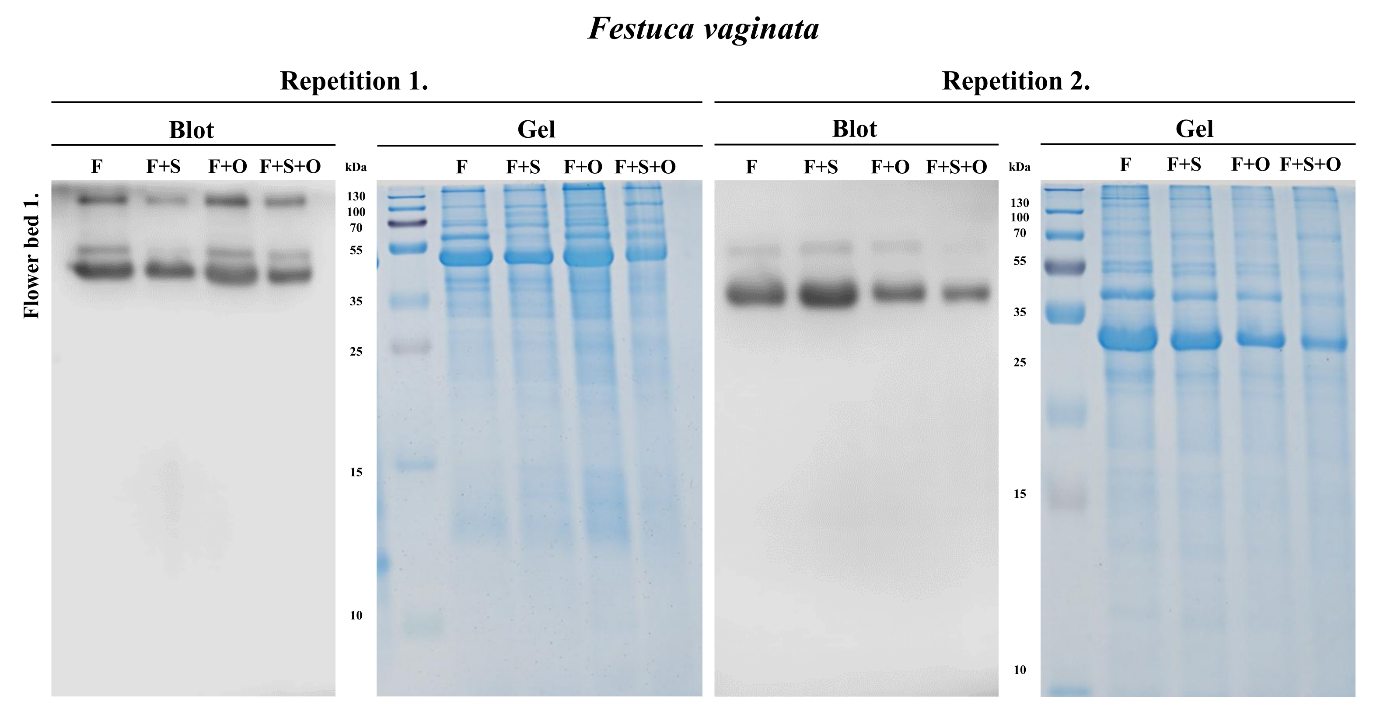


**Figure S4** Western blot gels of RUBISCO from *Festuca vaginata* leaves with and without the hemiparasite and its invasive alien competitor. F: *Festuca vaginata*, O: *Odontites luteus*, S: *Sporobolus cryptandrus*. Neither groups differed significantly from each other (see statistics in the main text). RUBISCO content decreased under parasite pressure but the competitor had no effect (see statistics in the main text).


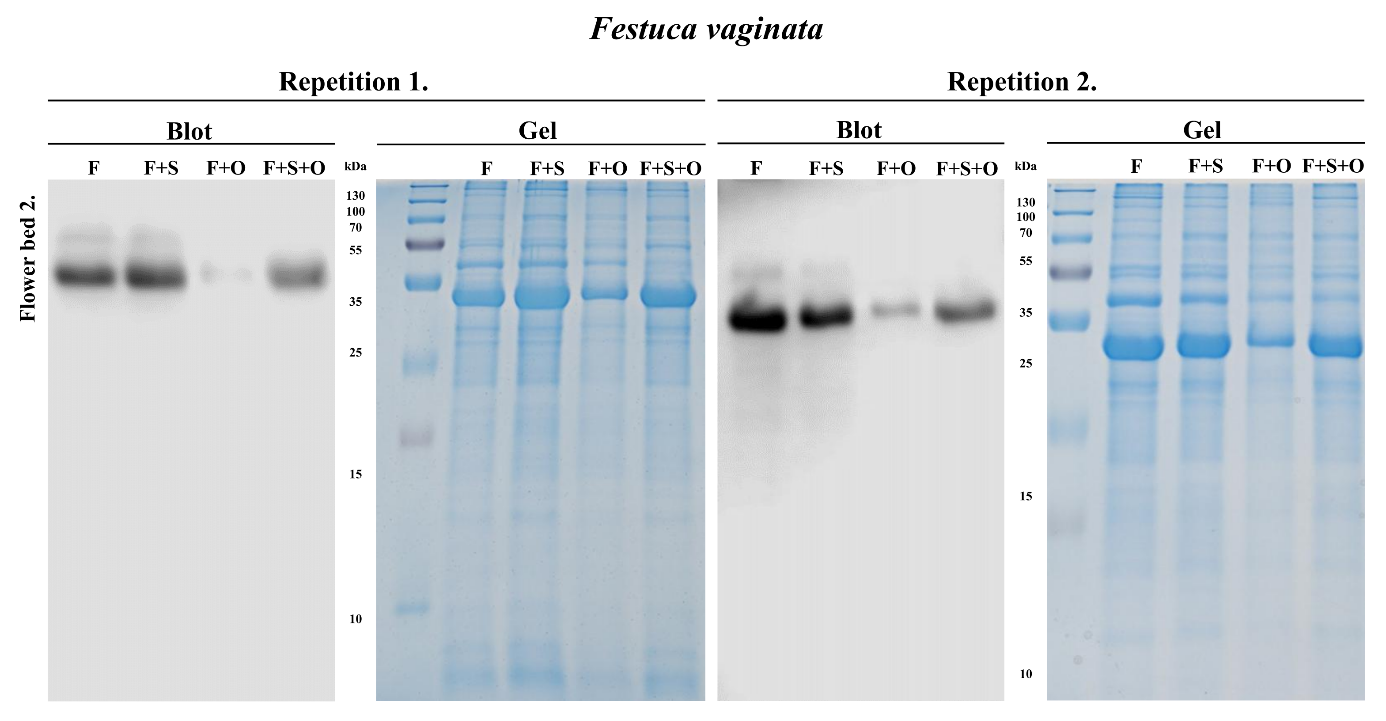


**Figure S5** Western blot gels of RUBISCO from *Festuca vaginata* leaves with and without the hemiparasite and its invasive alien competitor. F: *Festuca vaginata*, O: *Odontites luteus*, S: *Sporobolus cryptandrus*. Neither groups differed significantly from each other (see statistics in the main text). RUBISCO content decreased under parasite pressure but the competitor had no effect (see statistics in the main text).


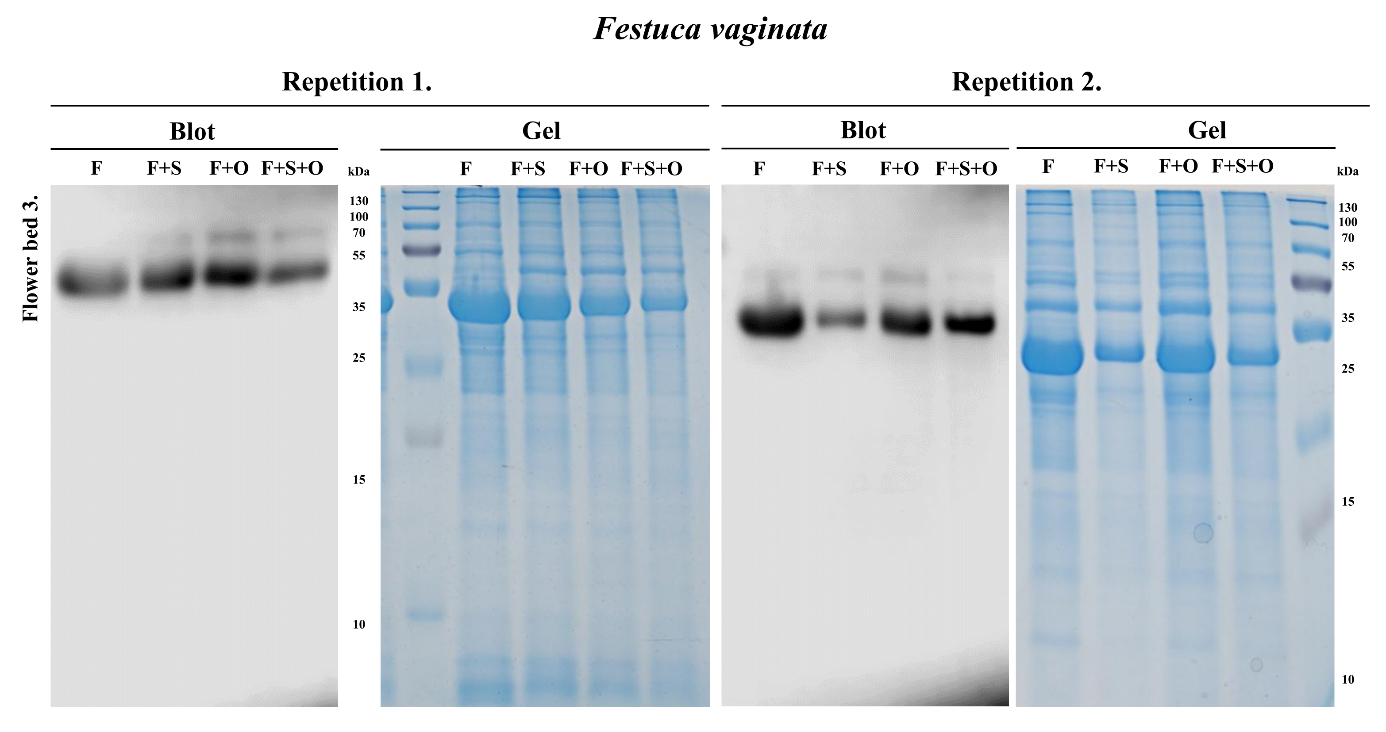


**Figure S6** Western blot gels of RUBISCO from *Festuca vaginata* leaves with and without the hemiparasite and its invasive alien competitor. F: *Festuca vaginata*, O: *Odontites luteus*, S: *Sporobolus cryptandrus*. Neither groups differed significantly from each other (see statistics in the main text). RUBISCO content decreased under parasite pressure but the competitor had no effect (see statistics in the main text).
